# Psoriasis: From Pathogenesis to Pharmacological and Nano-Technological-Based Therapeutics

**DOI:** 10.3390/ijms22094983

**Published:** 2021-05-07

**Authors:** Robert Gironés Petit, Amanda Cano, Alba Ortiz, Marta Espina, Josefina Prat, Montserrat Muñoz, Patrícia Severino, Eliana B. Souto, Maria L. García, Montserrat Pujol, Elena Sánchez-López

**Affiliations:** 1 Department of Pharmacy, Pharmaceutical Technology and Physical Chemistry, Faculty of Pharmacy, University of Barcelona, 08028 Barcelona, Spain; rgironpe7@alumnes.ub.edu (R.G.P.); acanofernandez@ub.edu (A.C.); albaortiz@ub.edu (A.O.); m.espina@ub.edu (M.E.); jprat@ub.edu (J.P.); mmunozjuncosa@ub.edu (M.M.); marisagarcia@ub.edu (M.L.G.); 2 Institute of Nanoscience and Nanotechnology, Universitat de Barcelona, 08028 Barcelona, Spain; 3 Center for Biomedical Research in Neurodegenerative Diseases Network, Carlos III Health Institute, 28031 Madrid, Spain; 4 University of Tiradentes (Unit) Av. Murilo Dantas, Aracaju 49010-390, Brazil; patricia_severino@itp.org.br; 5 Institute of Technology and Research (ITP) Av. Murilo Dantas, Aracaju 49010-390, Brazil; 6 CEB—Centre of Biological Engineering, Campus de Gualtar, University of Minho, 4710-057 Braga, Portugal; 7 Faculty of Pharmacy, University of Coimbra, Pólo das Ciências da Saúde, Azinhaga de Santa Comba, 3000-548 Coimbra, Portugal

**Keywords:** skin inflammatory diseases, psoriasis, psoriasis versus atopic dermatitis, biodegradable nanoparticles, microneedles, clinical trials

## Abstract

Research in the pathogenesis of inflammatory skin diseases, such as skin dermatitis and psoriasis, has experienced some relevant breakthroughs in recent years. The understanding of age-related factors, gender, and genetic predisposition of these multifactorial diseases has been instrumental for the development of new pharmacological and technological treatment approaches. In this review, we discuss the molecular mechanisms behind the pathological features of psoriasis, also addressing the currently available treatments and novel therapies that are under clinical trials. Innovative therapies developed over the last 10 years have been researched. In this area, advantages of nanotechnological approaches to provide an effective drug concentration in the disease site are highlighted, together with microneedles as innovative candidates for drug delivery systems in psoriasis and other inflammatory chronic skin diseases.

## 1. Introduction

Psoriasis is an inflammatory skin disorder that mainly depends on genetic predisposition and ageing. However, there are some environmental risk factors, such as trauma (e.g., Koebner phenomenon), infection, and drugs, that have been proposed to influence the development of this inflammatory skin disease. This pathology affects around 2% of the population worldwide, but it shows some variability, depending on the type of skin [1]. In this sense, in Caucasian and Scandinavian people, it increases its prevalence, rising until 11% of their population is affected [2]. Several types of psoriasis have been identified, depending on how it affects the patient. The majority of psoriasis cases correspond to psoriasis vulgaris or plaque-type psoriasis (almost 90%). Plaque-type psoriasis is easily recognised by the pruritic plaques covered in silvery scales [3]. Innate and adaptative immune responses are responsible for the development of psoriatic inflammation, while innate immune responses are more significant in plaque-type psoriasis [4]. One of the most accepted mechanisms involves an overexpression of antimicrobial peptides in psoriatic skin, acting as a trigger and a continued maintenance phase [5]. Some of the most studied hallmarks of psoriasis are LL-37, β-defensins, and S100 proteins [6]. In the earlier phase, there is an abnormal decease of LL-37 and different antimicrobial peptides from keratinocytes in stress conditions, such as physical injury. LL-37 is released by damaged keratinocytes and forms complexes with genetic material from damaged cells around the keratinocyte. LL-37 has been recognised as a participant in the pathogenesis of the psoriasis, due to the boundaries with DNA-stimulating toll-like receptor 9 in plasmacytoid dendritic cells [7]. There are two ways in which LL-37 affects the activation of plasmatic dendritic cells. On the one hand, the stimulated production of type I IFN, which promotes phenotypic maturation of myeloid dendritic cells, has an important role in Th1 and Th17 differentiation and function [6,7,8]. Th-17 cells are special populations of CD4+ T cells that produce IL-17, IL-22, IL-21, TNF-α, and other cytokines and express lineage specific transcription factor Retinoic acid receptor-Related Orphan receptor (RORC) [9,10]. The family of Th-17 cells includes several cell types, all of them expressing ROR-γt and IL-23R. In a study published in 2018, the authors determined the mRNA expression level of RORC in patients with psoriasis and found significantly higher gene expression of RORC in patients with psoriasis than in control patients, thus concluding that Th-17 plays a role in the pathogenesis of the disease [11]. RORγT and its isoform, ROR, are encoded by a single gene called Rorg (also known as Rorc). Both isoforms use the last 9 exons (exons 3–11) of the Rorg gene, but the other exons used by them are different. Consequently, the RORγT mRNA differs from that of ROR in the first 100 nt, which translates into distinct N-terminal amino acid sequences [12]. Expression of RORγt is not only confined to Th17 cells, but also regulates cytokine production in other cell types, such as CD8+Tc17 cells, invariant natural killer T cells, ILC3, and γδ T-cells [13]. All of these contribute to autoimmune tissue inflammation. Moreover, it has been found that RORγ-deficient mice show diminished Th17/IL-17 responses and are protected against autoimmune inflammatory diseases, such as psoriasis-like skin inflammation [14]. Pharmacological modulation of RORγt by low molecular weight inhibitors is therefore an attractive approach to inhibit the proinflammatory IL-17/IL-23 axis. Given the fact that it is a nuclear hormone receptor, the activity of RORγt is regulated in a ligand-dependent manner. Therefore, numerous inhibitors targeting the ligand binding domain (LBD) of RORγt have been reported recently. These drugs were effective in suppressing the IL-17 pathway and showed good efficacy in different inflammatory autoimmune disease rodent models [15].

Th-17 cells are activated by IL-6, IL-1β, and IL-23 and trigger chronic inflammation and autoimmunity, while TGF-β and IL-6 activated Th-17 cells are weakly pathogenic and are mostly involved in tissue integrity and defense [16]. On the other hand, LL-37 complexed with DNA or RNA stimulates plasmacytoid dendritic cells through both TLR9 and TLR7. Furthermore, there are slan+ monocytes secreting high amounts of (TNF)-α, IL-23, and IL-12, responding to the LL-37-RNA complexes. There is also migration of myocytic dendritic cells into lymph nodes with an extra proliferation of (TNF)-α, IL-23, and IL-12 [17].

**Figure 1:**
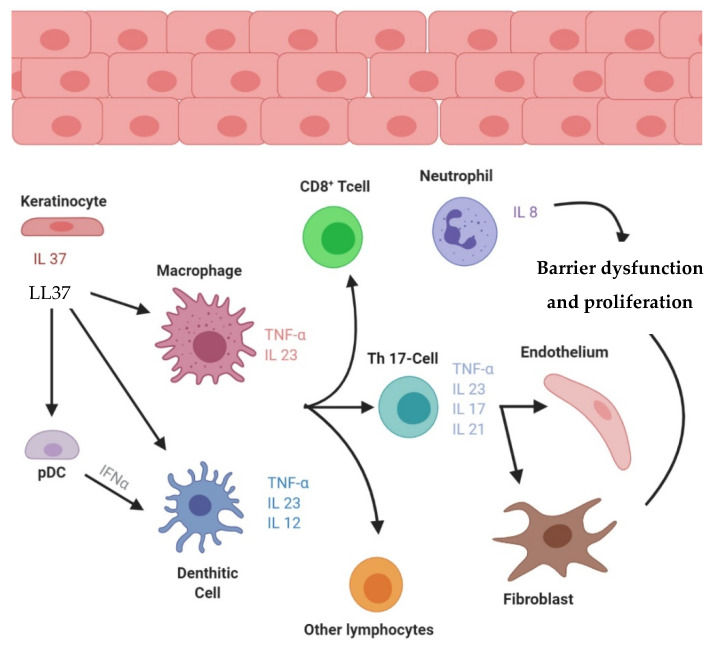
Plaque-type psoriasis pathogenesis principal hypothesis.

The maintenance phase of psoriatic inflammation is driven by the activation of the adaptative immune response via the T cell subsets [18]. The proliferation of keratinocytes in epidermis is engaged by two different vias, inflammation by action of TNF- α, IL-17 and IFN- γ, and LL-37 complexed with DNA, resulting in an increasing production of type I IFNs [4]. All these mediators further maintain keratinocytes activation, producing LL-37, proinflammatory cytokines (TNF-α, IL-1β, IL-6), chemokines, and S100 proteins, propagating the chronic inflammation. Altogether, these promote keratinocyte proliferation and production of AMPs and chemokines, which promote neutrophil recruitment and sustain skin inflammation [19]. Figure 1 depicts the plaque-type psoriasis pathogenesis main hypothesis.

Plaque-type psoriasis is characterised by the inflammatory pathway TNFα–IL-23–Th17. There are several types of IL-17, which are produced by different cell-types, such as hematopoietic cells, namely CD8+ T cells (Tc17), invariant NKT cells, γδ T cells, non-T non-B lymphocytes (termed type 3 innate lymphoid cells), and neutrophils. Inflammatory responses are regulated by IL-17A-F cytokines [20]. It has been stablished that the most important signalling in psoriasis is mediated by a receptor that could be activated by two different cytokines, IL-17A and IL-17F, IL-17A having a stronger effect [20]. Additionally, there is a recruitment of the ACT1 adaptor protein when IL-17A binds to the receptor complex, composed of IL-17RA subunits and one IL-17RC subunit.

There is an activation of some intracellular kinases with the interaction between ACT1 and the IL-17 receptor complex. These intracellular kinases include extracellular signal-regulated kinase (ERK), p38 MAPK, TGF-beta-activated Kinase 1 (TAK1), I-kappa B kinase (IKK), and glycogen synthase kinase 3 beta (GSK-3 beta). All these kinases enable pro-inflammatory cytokines, chemokines, and antimicrobial peptides. Th1 and Th2 cytokines act through Janus kinase (JAK)-STAT signalling pathways, whereas Th17 responses are mediated by ACT1 and NFκB [21]. Alternatively, γδ T cells are able to produce IL-17A independently of the IL-23 stimulus [22].

**Figure 2:**
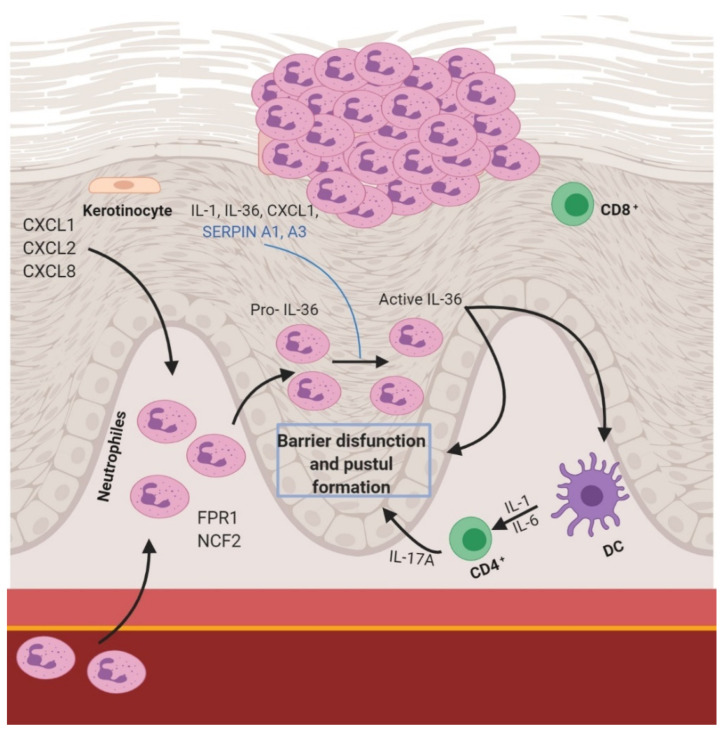
Pathophysiology in pustular psoriasis.

The second most common psoriasis type is pustular psoriasis, which is characterized by multiple coalescing sterile pustules. Whilst in plaque psoriasis, the adaptative immune system has a greater importance in the pathogenesis, obtaining good results with therapies targeting these elements [23,24]. It seems that the innate immune system plays an important role in pustular psoriasis [8] and those therapies used in plaque psoriasis are less effective [25]. Although there is an overlapping of some metabolic paths, there are significant differences in the general pathogenesis of these psoriasis types. It seems that GPP principally depends on the activities of KCs, neutrophils, and monocytes [26]. There is an increased expression of IL-1β, IL-36α, and IL-36γ in pustular psoriasis than in plaque psoriasis due to a mutation in gene IL 36 RN [26]. This overexpression of IL-36 appears to be the central mechanism that promotes neutrophil accumulation in the epidermis [27,28] (Figure 2). The significant presence of neutrophil chemokines CXCL1, CXCL2, and CXCL8 (IL-8) is in accordance with the assumed pathogenesis of GPP [26].

Generalized pustular psoriasis presents with an acute and rapidly progressive course, characterized by diffuse redness and subcorneal pustules, and is often accompanied by systemic symptoms [29]. Another rare type is guttate psoriasis, which is known for its expression by small erythematous plaques and for being mostly common in children and teenagers [30]. There are no data that show any difference in the pathophysiologic mechanism from plaque-type psoriasis. It has been proposed that some streptococcal superantigens stimulate the proliferation of T cells in the skin in guttate psoriasis [31]. It is known that there is some homology between streptococcal proteins and human IL-17 keratin proteins. There may be an important role played by molecular mimicry in patients with the major histocompatibility HLA-Cw6 allele, since CD8(+) T cell IFN-γ responses were elicited by K17 and M6 peptides in said patients [18,32]. One less common type is inverse psoriasis, which is characterized for affecting intertriginous locations and by being more erosive than erythematous plaques of type-plaque. As no data exist showing any difference between the pathophysiologic mechanism of common psoriasis and inverse psoriasis, it seems there is a decrease in the number of CD161+ cells in the plaques of inverse psoriasis. This is speculated to be due to the constant microbial colonization of those areas affected by inverse psoriasis [33,34]. Finally, the most severe type, erythrodermic psoriasis, is an acute condition, in which most of the body surface is erythematous and inflamed. There are no data that show any difference in the pathophysiologic mechanism of common psoriasis versus erythrodermic psoriasis [2].

## 2. Methods

For the literature research, different databases have been used (SciELO, Springer link, MEDLINE, Embase, LILACS, and PubMed), searching for articles published over the last 10 years. Several trials registered at the US Food and Drug Administration (FDA) and European Medicines Agency (EMA) were also researched. As the keywords, the following terms were used: psoriasis; nanoparticles and psoriasis; microneedles and psoriasis; skin inflammatory diseases; clinical trials and psoriasis; cytokines and psoriasis.

## 3. Current Marketed Therapies

As psoriasis is a chronic disease, long-term therapy is usually necessary. Depending on different factors, such as disease severity or comorbidities, there is a wide range of treatments that have different responses depending on the patient. There are different grades of psoriasis, measured by different factors, such as severity of lesions, percentage of affected surface area, and quality of life [35]. One of the most widely used criteria is the Psoriasis Area and Severity Index (PASI), being the most accurate. The PASI allows comparisons between clinical trials and objectively evaluates the effectiveness of different antipsoriatic drugs.

Following these criteria, there are several grades of psoriasis, from mild to moderate to severe psoriasis. Almost 80% of psoriasis patients have mild to moderate psoriasis, which can be treated with topical treatments [36,37]. In moderate cases, there are some topical treatments, based on corticosteroids, used in combination with other drugs, such as vitamin D, or alone, such as vitamin D derivatives, vitamin A, and anthralin, which are examples of actual topical treatments.

Vitamin D3 analogues constitute the first line topical treatment for plaque psoriasis and moderately severe scalp psoriasis [38,39]. The beneficial effects of vitamin D induced by exposure to sunlight in the treatment of psoriasis has been known for decades [40,41,42]. However, vitamin D has shown a relevant yet controversial role in psoriasis, as well as other skin diseases [43,44]. In this area, Filoni et al. aimed to shed some light by confirming reduced vitamin D levels in psoriatic patients and stablishing a relationship between vitamin D levels and psoriasis length [45]. Moreover, Lee et al. also confirmed lower presence of 25-hydroxyvitamin D (25OHD) in psoriasis patients, 25OHD being an possible indicator of the amount of stored Vitamin D [46,47]. However, there is little evidence that the increase in 25OHD after phototherapy correlates with improved disease severity [48].

**Figure 3:**
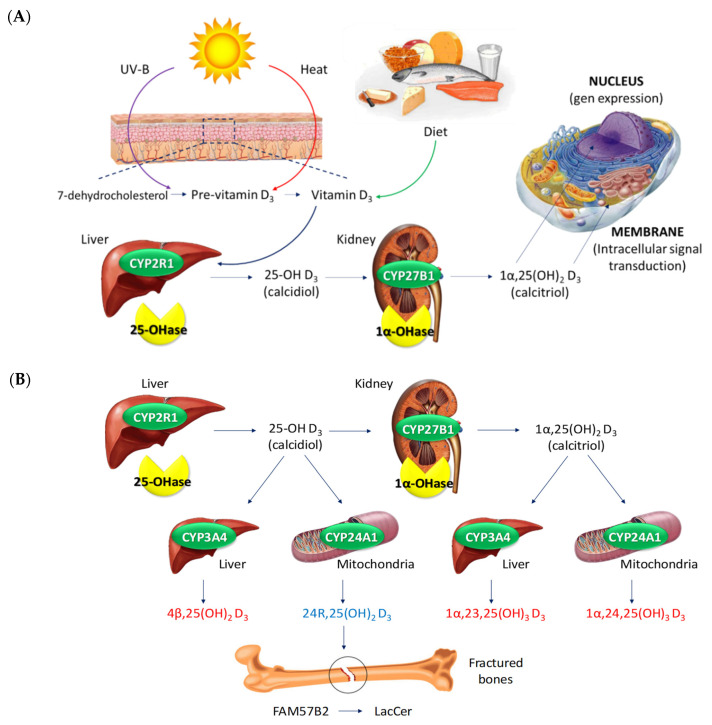
The physiological pathway of vitamin D synthesis and activation. (**A**) Ultraviolet radiation promotes the conversion of 7-dehydrocholesterol to pre–vitamin D_3_, which isomerizes to vitamin D_3_ (also called cholecalciferol) in the skin, due to sun heat. Diet supplements directly provide Vitamin D_3_, also called ergocalciferol. In the liver, a 25-hydroxylation by CYP2R1 is carried out, thus leading to the formation of 25-hydroxyvitamin D_3_. In the kidney, CYP27B1 further hydroxylates 25-hydroxyvitamin D_3_ at the 1-α position, resulting in the formation of the active hormone 1α,25-dihydroxyvitamin D_3_. The active form of vitamin D then enters into the cell via diffusion or endocytic receptor for transcription. Inside the cell, vitamin D binds to both vitamin D receptors at the nucleus and cell membrane. In the nucleus, both active forms of vitamin D and its receptor form a regulatory complex that finally leads to the beginning of the transcription process. In the cell membrane, binding to vitamin D receptors lead to several intracellular signal transductions. (**B**) An alternative pathway has been described. In this case, 25-hydroxyvitamin D_3_ and 1α,25-dihydroxyvitamin D_3_ metabolites are hydroxylated by other two cytochromes: on the one hand, a dominant gain-of-function mutation in CYP3A4, mainly located in the liver, leads to acceleration in vitamin D inactivation; on the other hand, hydroxylation by CYP24A1, mainly located in the mitochondria, gives rise to the formation of an active metabolite, 24R,25(OH)_2_D_3_. This molecule has been described to bind to FAM57B2 in fractured bones. This leads to the production of lactosylceramide (LacCer), which is essential for the callus formation and fracture healing.

Moreover, Vitamin D is involved in the proliferation of keratinocytes (Figure 3). In fact, the precursor of vitamin D, 7-DHC, is localized in the keratinocyte membrane, and, by the UVB activation, it is transformed into pre-vitamin D3 or cholecalciferol, which is later converted first to 25OHD by the enzymes CYP27A1 and CYP2R1 and then to 1,25(OH)D or calcitriol, the active form of vitamin D [1,49,50,51,52]. Calcitriol regulates differentiation and proliferation of keratinocytes, as well as the balance of the cutaneous immune system and cellular apoptosis. Interestingly, at low vitamin D concentrations, a promotion of keratinocyte differentiation is found, whereas, at high concentrations, an inhibitory effect occurs [43]. Due to the regulation of calcium exerted by vitamin D through calcium receptor induction and phospholipase C enzymes, calcitriol and its analogues (calcitriol, calcipotriol, tacalcitol, hexafluoro-1,25(OH)D, and maxacalcitol) have demonstrated interesting features. In fact, in vitro studies demonstrated that they were able to reduce the psoriatic upregulated levels of S100A7 and regulate cell proliferation in the stratum basale in addition to increasing keratin synthesis and regulating glycoceramides production. Therefore, a decrease in calcitriol or a loss of function of its receptor causes epidermis disruption that results in hyperproliferation of the basal layer [43]. The anti-inflammatory effect attributed to vitamin D may also result from inhibition of production of IL-2, IL-6, and interferon-gamma (IFN-γ). Furthermore, topical calcipotriol inhibits human beta defensin and proinflammatory cytokines, which are increased in psoriatic lesions [43]. Moreover, calcitriol, as well as novel vitamin D3 derivatives, inhibit the transcriptional activity of NFkappaB, a major inducer of inflammation [41].

Interestingly, several studies identified an association between polymorphisms of vitamin D receptor (VDR) and psoriasis susceptibility [43]. However, this is still controversial [53,54]. The mechanism of vitamin D is mediated by the vitamin D receptor (VDR), and after its activation, it interacts with retinoid X receptor (RXR) to form a heterodimeric complex. The VDR-RXR complex is recruited to the vitamin D response elements (VDREs) in the promoter of target genes to regulate their expression. This process is described as the genomic action of vitamin D in contrast to the nongenomic action, which is the direct effect that vitamin D has on the previously mentioned signalling pathways [38]. Allelic variations in individual VDR genes may determine a different response to treatment: the isoform A of VDR is associated with a greater therapeutic response in psoriatic patients [43]. In this sense, VDR ligands inhibit the expression of pro-inflammatory cytokines produced by T lymphocytes, which are responsible for the exacerbation of the skin inflammation. Apart from that, 1α,25(OH)2D3 enhances expression of IL-10 within the psoriatic lesions. Moreover, biological activity of vitamin D3 analogues leads to suppression of the T cell-mediated immune response [41]. In addition, there is evidence that one active form of vitamin D3 synthetized by CYP11A1, 20(OH)D3, has anti-proliferative, pro-differentiation, and anti-inflammatory effects on cultured skin cells, comparable to or better than those of 25OHD. Thus, it has been proposed as a new candidate for primary or adjuvant therapy of hyperproliferative or inflammatory disorders, such as psoriasis [55].

As it has been mentioned, apart from the classical activation route for vitamin D, alternative routes have been described, such as the CYP11A1 route, which leads to hydroxy metabolites. The traditional role of CYP11A1 was associated to initial steroid synthesis, solely in steroidogenic organs using cholesterol as the substrate [55]. This involved hydroxylations at C22 and C20, followed by oxidative cleavage of the bond between C20 and C22 to produce pregnenolone, a precursor to all steroids [55]. However, it has now been documented that alternative substrates from cholesterol have been identified, such as 7DHC, vitamins D2 and D3, ergosterol, and lumisterol [56]. It has been dilucidated that CYP11A1 initiates the metabolism of vitamin D: from D3 to (OH)nD3 [56]. The main metabolite resulting from a single hydroxylation of D3 by CYP11A1 is 20(OH)D3, but 22(OH)D3 and 17(OH)D3 are also produced [57]. The major dihydroxy and trihydroxy metabolites formed from CYP11A1 hydroxylation of 20OHD3 include 20,23(OH)2D3, 20,22(OH)2D3, 17,20(OH)2D3, and 17,20,23(OH)3D3 [57]. Moreover, further hydroxylation of CYP11A1-derived metabolites can occur by CYP27B1, CYP24A1, and CYP27A1 [58].

Apart from the well-known mechanism by binding 1α,25(OH)2D3 with VDR, there are different nongenomic associated sites. VDR has an alternative form binding-A-pocket, which leads to rapid nongenomic responses at the cell membrane level [55]. Furthermore, there is a rapid steroid binding protein disulphide-isomerase A3 (PDIA3), which has been identified as an alternative membrane-bound receptor. PDIA3 activates phospholipase C in a G protein-coupled process and results in the production of inositol trisphosphate (IP3) and diacylglycerol. These two cellular messengers mediate the rapid release of calcium from the cellular stores [59]. Recently, RORγ, another nuclear receptor, has been identified as a target for vitamin D, where D3 hydroxyderivatives could act as antagonists, regulating some phenotypic expressions with affectation in several immune functions, metabolism, and cerebellar development [59]. Recently, Vitamin D has been studied outside the immune system, with some VDRs localised in the intestinal barrier, regulating intestinal inflammation, autophagy, or gut microbiota [60].

In severe cases, a systemic treatment, sometimes combined with local treatment, is necessary due to the increased extension of the affected surface. Some strategies for enhancing the therapeutic results of the topical treatments, such as using some adjunct agents, such as penetrating or permeating enhancers, or phototherapy, can be employed [61]. It has been demonstrated that monotherapies, which require irregular applications, are not the best options for the optimisation of the treatment adherence [62]. One option for improving the treatment adherence is a combination regime [63], which allows applications in a fixed dose with a low frequency of application [64].

Topical therapies are the backbone of management of psoriasis. They are safe and well-tolerated by the patients. Currently, vitamin D derivatives are used in combination with betamethasone for mild plaque-type psoriasis cases. Moreover, topical calcineurin inhibitors (TCIs) and Vitamin D analogues are the treatment of choice among the various topical agents available for different subtypes of psoriasis. For example, TCIs can be used as steroid-sparing agents on the face and intertriginous areas in inverse psoriasis [65]. Tazarotene can be used as an effective maintenance therapy (Table 1). New vehicle formulations, such as gels, lotions, solutions, shampoos, foams, etc., have been developed to improve physicochemical properties, such as lack of adherence, penetration rates, or different pharmacological forms, depending on the application site, thus improving the patient compliance, which is of utmost importance for optimum results. Target-based topical agents are being developed and tested. Moreover, advancement in nanotechnology has led to the possibility of improving the efficacy of topical agents through targeting, improving adherence of pharmaceutical dosage forms and/or drug penetration rates, and minimizing side effects. The formulation of newer molecules and newer drug delivery systems will significantly expand the therapeutic stock for the treatment of psoriasis. As long as there is a general consensus about using topical therapy for mild to moderate psoriasis, there are not a great number of studies about the long-term use of these medicines.

There is a wide range of treatments for severe psoriasis, from topical gels to systemic oral drugs. There are some different types, with different targets, having a wide range of effectiveness and safety. On the one hand, there are the oldest class-level treatments [66], such as acitretin, ciclosporin, fumaric acid, esters, and methotrexate. On the other hand, there are the biologics: anti-TNF alpha treatments, such as etanercept, infliximab, adalimumab, and certolizumab. All of these systemic drugs have been approved for their use in psoriasis treatment. It has been demonstrated by different studies that biologics outperform the small molecules to reach PASI 90 (Table 2).

Despite advances in the effectiveness of this different systemic treatments, they do not work in all patients, showing problems associated with thereproducibility of results. It seems that low absorption of treatments and the possible toxicity of the drugs are common and there are relevant drawbacks to guaranteeing an effective treatment. Thus, there are still investigations finding different drugs and biosimilar agents for new treatments [100]. Furthermore, there is an increasing interest in the study of different administration routes and new carriers for these drugs, such as microneedles or the use of nanotechnology.

## 4. Clinical Trials

Research in new pharmacological approaches for psoriasis has been intensely carried out during the last decade. In recent years, many breakthroughs in biological treatments and new small molecules have been developed. Furthermore, there are different drugs that are under clinical trials. From topical application to oral administration, there are cutting edge drugs that have been used to improve actual pharmacological arsenal for psoriasis. There are some new drugs in different types of administration are still in clinical trials at the date of this review but might be approved in the coming months (Table 3).

Topical application is the main administration route for psoriasis-targeted drugs. As has been specified before, topical formulations are the backbone of the treatments for psoriasis, especially in moderate and mild cases. This is why there is important interest on the development of new formulations or new active drugs that could improve the performance of existing treatments. In fact, there are many drugs in topical formulations under clinical trials. Parenteral administration has led to different approaches in psoriasis. Despite the fact that the majority of biological treatments released in recent years have been formulated as subcutaneous medications, oral administration is the most explored in the current clinical trials.

## 5. Nanotechnological Approaches for Psoriasis

In the past 20 years, there has been a revolution in different fields related to the use of nanotechnology. There has been an intense study of nanoparticles for the design and manufacturing of new treatments in medicine. Thanks to the versatility of this technology, it is possible to use a wide range of materials with completely different properties in different functions [126]. Nanoparticles are mainly used as carriers for chemotherapeutic active drugs. Therefore, biodegradable nanoparticles constitute one of the most relevant approaches for the present and future treatment of psoriasis.

### 5.1. Polymeric Nanoparticles (PNPs)

PNPs are used as biomaterials due its characteristics, such as good biocompatibility, wide range of sizes and structures, bioimitative characteristics, and simple elaboration. It is possible to derivatize their structure on many paths, permitting good targeting as a drug delivery system. There are many types of polymer-based nanoparticles—the main types are nanospheres and nanocapsules—but there are many others, such as dendrimers and micelles.

#### 5.1.1. Nanospheres

Nanospheres are based on the homogeneous dispersion of the drug inside their polymer matrix structure [121]. The main objectives of nanospheres, which could be biodegradable or non-biodegradable depending on the materials employed on their manufacturing, are to enhance solubility, improve absorption, and control drug release.

Batheja et al. prepared tyrosine-derived nanospheres (tyrospheres), loaded in a gel, obtaining significant results, demonstrating an improving absorption in studies in vitro and in vivo [127]. In this study, there was a comparative difference between different gels for supporting the nanoparticles. The gel type had a significant effect on the nanosphere dispersion: nanospheres were more homogeneously dispersed in the METHOCEL™ K15M0 (HPMC) than in Carbopol. Three concentrations (3, 6, and 10 mg/mL) of NSP dispersions in HPMC were tested, with 3 mg/mL resulting in the most homogeneous dispersion of individual particles with ~40 nm in diameter, which is similar to the previously reported size in bibliography. Moreover, the use of tyrospheres as a cargo system for the delivery of Vitamin D3 treatment has been studied by Diering et al., obtaining different particle sizes from 64.3 to 73.4 nm, depending on the concentration of Vitamin D3 [128]. Their conclusions evidence that there was an enhanced absorption of the treatment, having an increasing concentration of the drug compared to the control.

#### 5.1.2. Nanocapsules

Nanocapsules are nanoparticles, with a structure based on the encapsulation of the drug in the core of the nanostructure, protected by the polymer shield [121]. Its core can consist of an oily phase or a polymer matrix, where the drug is dissolved, or the drug can be in its molecular form, distributed homogeneously in the nanoparticle. The main advantages offered by nanocapsules include sustained release, incremental drug selectivity, and improvement of drug bioavailability and alleviation of drug toxicity [129,130].

In this context, Marchiori et al. worked in dexamethasone-charged nanocapsules for drug delivery through topical administration [131]. The composition of nanocapsules consisted of an oily phase in the core of capric triglyceride mixture and a shield of polycaprolactone in a Carbopol gel, obtaining a particle size of 201 nm with an encapsulation rate of >95%. Its study in vitro demonstrated a controlled release of the drug with a good stability, suggesting further studies in vivo in the near future.

#### 5.1.3. Dendrimers

Dendrimers are a type of nanoparticle, characterised by being spherical, monodisperse and multivalent macromolecules. Their structure allows some unique features, such as their good solubility, biocompatibility, and a good reactivity ratio [132]. Moreover, depending on which structure is designed, the drug delivered could be encapsulated or as a covalently conjugated drug form, preventing any chemical or biological degradation. In addition, their structure permits the use of different drug release mechanisms [133].

Agrawal et al. studied the ability of polypropylene imine dendrimers to transport dithranol for topical treatment [134]. Its formulation obtained a particle size of 8 nm, with the drug loaded in the dendrimers after an exposure of dendrimers to dithranol. There was a significant improvement in drug permeation, from 35% to 95% of drug penetration, and a decrease in skin irritation, compared to a solution of dithranol. Conclusively, dendrimers should be studied as possible candidates in drug delivery systems in the future.

#### 5.1.4. Micelles

Micelles are nanocarriers that are constituted by amphiphiles, having a concentration above critical micelle concentration, providing a core corona structure. Due to its composition, micelles are good protectors for aquaphobic drugs, which are b protected inside the micelle and surrounded by a hydrophobic barrier [135]. Meanwhile, in the external face, there is a hydrophilic barrier, allowing the micelle to be solubilized in aqueous media. This structure permits an elongation of the drug circulation life, increasing the permeation and absorption of the drug. Micelles have many advantages, such as high drug loading, increased bioavailability, low degradation of drugs, and decreased side effects. All its characteristics make micelles good candidates for topical treatments in skin diseases, such as psoriasis. In this context, Lapteva et al. studied MPEG-dihex PLA as a delivery system for tacrolimus, in which micelles diameters were below 50 nm [136]. This study was encouraged by the fact that tacrolimus is not soluble in water, being a good candidate for an improvement of solubility, bioavailability, and permeability. In the in vivo studies, micelles remained on the skin surface, unable to pass to the stratum corneum but remaining in the follicular ducts. Conclusively, micelles were not able to enhance permeation through the skin, which makes them less effective than other drug delivery systems.

### 5.2. Lipid-Based Nanoparticles

Lipid nanoparticles are produced with physiological lipids, which provide safety and non-toxicity from the structure of the nanoparticles [137,138]. Due to its structure, lipid nanoparticles have several advantages, such as an enhanced stability of the drug, thus a longer lifetime in circulation, biodegradability, targeting, and good drug load, with very competitive costs.

#### 5.2.1. Liposomes

Liposomes are closed vesicles, produced by one or more lipid layers overlapped from the core to the surface, having the same number of aqueous compartments between layers [137,139]. Liposomes are one of the most studied lipid-based nanoparticles in the treatment of dermatological disorders due to their unique features of being able to act like organic solvents to solubilise non-soluble drugs, getting in groups acting as local depots for a control releasing of the drug, enhancing penetration and diffusion of molecules with low solubility into the lipid covering of the stratum corneous [139]. Additionally, liposome physicochemical characteristics are easily controlled by their composition. Features, such as charge, size, permeability, or durability, are malleable by researchers, giving different approaches for the same treatments.

Wadhwa et al. studied the use of a loaded liposome of fusidic acid for a proficient treatment of plaque psoriasis [140]. These liposomes where prepared, obtaining a range in different physicochemical characteristics depending on the concentration parameters, such as size, ranging from 572.7 to 740.1 nm, or entrapment efficiency, ranging from 52.1% to 72.6%. This study showed that the formulations were stable and had an enhancing effect on the efficacy of the anti-psoriatic drug compared with a conventional preparation in in vivo studies in mouse tail models. In this sense, liposomes are effective for their use in a drug delivery system in skin disorders, such psoriasis.

#### 5.2.2. Lipospheres

Lipospheres are lipid-based nanoparticles, which are produced by a stabilization of an aquaphobic core by coating phospholipid molecules on its surface. This structure provides some features to lipospheres that could not be compared with other lipid-based nanoparticles, such as its low costs, an increased stability, easy production, controlled release rate, and a good dispersion rate in aqueous media [141]. Lipospheres have been used in different administration routes, such as oral, intravenous, or transdermal, with good results in the treatment of some diseases, including psoriasis.

In psoriasis treatment, Jain et al. used tacrolimus and curcumin loaded in a liposphere gel formulation and studied its effectiveness in psoriasis patients compared to a control formulation of both drugs in a solution [142]. Lipospheres of 47 nm were produced and incorporated to topical gel formulation. Furthermore, there was a study of distribution of the formulation, observing an improvement on the permeation in the different layers of the skin in mouse models. In addition, there was a study of the effectivity of the treatment compared to the use of the curcumin and tacrolimus solution, being observed as an enhanced performance and histopathological improvement with the lipospheres formulation. Moreover, there was a notable decrease in the levels of TNF-α, IL-22, and IL-17 compared to actual treatment, suggesting that the use of lipospheres could be interesting in the development of new treatments for psoriasis.

#### 5.2.3. Ethosomes

Ethosomes are vesicular carriers based on ethanol, phospholipids, and water for topical application. Their composition permits good skin penetration, due to their softness and flexibility, caused by the high ethanol concentration. The proposed mechanism for penetration enhancement by ethosomes is based on the dual effect of ethanol on both the lipid bilayers in the stratum corneum and in the vesicle: ethanol enables fluidization of the lipids in the ethosomal structure on one side, along with changes in the arrangement of the lipids in the skin barrier on the other side. This allows the soft vesicles to penetrate the altered structure of the stratum corneum and release the activity in the deeper layers of the skin [143].

In this area, Zhang et al. prepared ethosomes as a delivering system for psoralen, obtaining a particle size ranging from 56.71 nm to 159.07 nm, showing the best entrapment efficiency ethosomes around 150 nm [144]. They studied the permeation of the drug compared to an ethanolic tincture of psoralen, observing a notable improvement (almost 7-times fold) in the deposition of the drug in rat skin. Conclusively, it was stated that the permeation and the penetration in the skin using ethosomes could decrease toxicity and increase the therapeutical effects in long-term therapy for skin disorders like psoriasis.

#### 5.2.4. Solid Lipid Nanoparticles

Solid lipid nanoparticles (SLNs) are one of the newest lipid-based nanoparticles, formed by a blend of physiological lipid and surfactants [145]. These SLNs could be formed in different ways; the aquaphobic drug moiety would form them in a lipid medium or it could be dispersed to create a drug layer surrounding the lipid core [145]. Due to its composition, SLNs have many advantages, in comparison with other topical vehicles, such as controlled drug release, a decreasing of skin irritation, and protection for the active drugs. Moreover, their size permits an improvement in the permeation of the drug across the skin due to close contact between the nanocarrier and the stratum corneum [146,147]. Because of their characteristics, there is an increasing interest in their use for topical treatments in diseases like psoriasis [148].

In this context, Pradhan et al. determined the potential of SLNs for being used as a delivery system for a prolonged release of fluocinolone acetonide [149]. The nanoparticles were selected after an optimization process based on some indicators, such as size and drug loaded, which were determined by X-ray diffraction and a transmission electron microscope. The optimized formulation was achieved, with the formulation composition having 2% (*w*/*v*) lipid concentration, 1% (*w*/*v*) surfactant, and 0.06% (*w*/*v*) drug concentration. Particle size and entrapment efficiency of the optimized formulation were 107.4 nm 87%, respectively, which represented a good agreement with the predicted values. There was a controlled prolonged release obeying Higuchi kinetics of drug release, whilst, in the suspension of the drug, there was a much quicker release, obeying zero order kinetics on skin models in vitro [121]. Furthermore, an increased concentration of fluocinolone acetonide on epidermis compared with the drug suspension was demonstrated. To sum up, it was demonstrated that the use of SNLs may be interesting in the development of new drug delivery systems to treat psoriasis.

#### 5.2.5. Nanostructured Lipid Carriers

Nanostructured lipid carriers (NLCs) are an innovating nanocarrier produced from a blend of solid lipids with spatially incompatible liquid lipids [150,151]. NLCs have many advantages from other formulations, such as an increased drug loading, a controlled release of the drug, improved biocompatibility, enhanced bioavailability, and the possibility of being administrated by different routes, such as oral, pulmonary, intranasal, or topical. Demonstrated was a huge potential for NLCs as a drug delivery system in topical formulations, due to its enhancing penetration ability, which permits an increase of bioavailability.

Furthermore, there is an occlusion property of the NLCs, which make them unique, not allowing transepidermal water loss, increasing skin hydration by the formation of a monolayered lipid film over the surface of the skin [152]. These features have awakened some interest in the use of NCLs in new treatments for skin diseases like psoriasis.

Avasatthi et al. developed a nanostructure lipid carrier loaded with methotrexate, one of the most widely used drugs for psoriasis, in a nanogel formulation [153]. The optimized formula was obtained using Precirol ATO 5, resulting in methotrexate-NLC, with an average particle size of 278 nm, a polydispersity index of 0.231, and entrapment efficacy of 22.3%. Effectiveness of this formulation was studied in vitro and in vivo, using the PASI score and an evaluation of histopathological characteristics. It was demonstrated that the use of the nanogel formulation had an increased effectiveness in the PASI score and had a prolonged life of circulation after 48 h of application. These results are relevant because they highlight the possibilities and potential of NLCs for new treatments for psoriasis.

### 5.3. Microneedles

**Figure 4:**
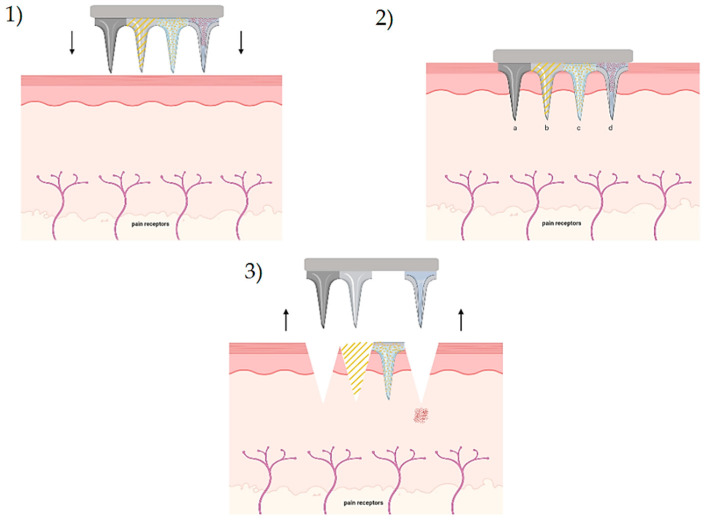
(**1**) Application of microneedles, (**2**) insertion, (**3**) extraction. Types of microneedles: (a) solid microneedles, (b) coated microneedles, (c) dissolving microneedles, and (d) hollow mi-croneedles.

Microneedle is a new drug delivery system, which consists of a base patch with perpendicular microneedles attached to bypass the stratum corneum. It is considered an intradermal drug delivery system, a new pharmaceutical form for psoriasis treatments. The patch has two parts; a base plate and the microneedles, which could be made of the same material or not. Microneedles must be strong and tough enough to perform their purpose with good mechanical resistance [154]. Moreover, the length of microneedles is between 25 to 2000 microns to assure their trespassing to the stratum corneum, but this is not long enough to activate the pain receptors in the skin, as shown below in Figure 4.

Microneedles constitute an elegant solution to increase drugs bioavailability. The mechanism of this delivery system consists of an attachment of the microneedle patch on the skin, permitting a micro-sized channel through the skin layer. The main objective of the delivery system is helping to trespass the first barriers of the skin, allowing the delivered drug to deposit directly into the stratum corneum. This procedure improves the permeability capacity of the drug, enhancing bioavailability [155]. Due to the increasing interest in the use of microneedles, different types have been developed, with some differences in their mechanism and materials.

There are four general types of microneedles: solid microneedles, coated microneedles, dissolving microneedles, and hollow microneedles. The only type that has been studied for psoriasis is dissolving microneedles.

Dissolving microneedles (DMNs) are produced with biodegradable materials, allowing them to dissolve themselves once they are in the skin [156]. The loaded drug in the needles is dissolved in the matrix of a polymer. The most important feature for the use of these microneedles is the material that the needles will be made of, which has to be strengthened enough to permit the perforation of the surficial layers of skin and show biodegradability without showing any toxicity. It must be considered that the material of the needles will be in the system of the patient. Therefore, microneedles have to be dissolved, circulate through the human body, and metabolized. One interesting feature of this type of microneedle is that drug release can be controlled by choosing the materials for the needles. Table 4 summarizes examples of biodegradable nanotechnology-based treatments for psoriasis.

Tekko et al. studied the combination of nanotechnology and microneedles using nanocrystals loaded with methotrexate disodium [170]. Methodology, optimization, and characterization of the microneedle’s patches with the nanocrystals were studied. The next step was studying their behavior in vitro, with interesting results on the release rates. Finally, an in vivo study was carried out with Sprague Dawley rats, comparing the use of microneedle patches with the usual administration route of methotrexate via oral administration. Their results suggest a successful insight into the use of microneedles in the delivery of methotrexate in rats, obtaining a sustained release of methotrexate during 72 h due to the drug retention capacity of the skin. Following these results, more studies are required to determine the efficacy of this new administration route in psoriatic models.

At present, there are still few studies that have used microneedles for drug delivery in psoriasis. Lee at el studied the safety and efficiency of using hyaluronic acid-dissolving microneedles with a cargo of methotrexate [171]. After studying the effects on 10 patients for 4 weeks, patient satisfaction was positive in general and there was a decrease in the PASI scores of their lesions. To sum up, an enhanced effectivity on the treatment was demonstrated, having a remarkable clinical effect on the study. However, more studies are necessary to confirm the data in this area, due to its small sample size and the lack of control groups.

## 6. Conclusions

Conventional drugs (i.e., commonly accepted as standard therapy or old class-level treatments), either administered by the oral or topical route, are still the backbone of the treatment of psoriasis. Nevertheless, we have witnesses emerging efforts towards the development of drugs and biosimilars with improved therapeutic outcomes, with less side effects and higher effectiveness. It is also true that new approaches, such as nanoparticles, have been proposed to promote new treatments. For this purpose, a wide range of nanoparticles is currently available, all of them with advantages and limitations. Nanoparticles based on a polymeric matrix, despite providing a controlled release of the loaded drug and improved permeation rates, may depict limited drug loading capacity and difficulties in scaling up the production lines. On the other hand, lipid-based nanoparticles may offer higher load capacity and encapsulation efficiency, in particular for poorly water-soluble drugs, and are biocompatible and may even act as absorption/penetration enhancers, given their lipid composition. Microneedles offer new solutions to the problems of conventional drugs, such as adverse-side effects, low bioavailability, or non-specificity in oral systemic treatments. Additionally, microneedles enhance permeation and increase specificity more than conventional topical treatments of different skin disorders. Despite the fact that only one type of microneedle has been applied in psoriasis treatment (dissolving microneedles), their results are really promising. These outcomes confirm that the use of this technology could be useful in the development of new drug formulations. To conclude, novel technologies, such as nanoparticles and microneedles and their combination, open a window to different perspectives to obtain innovative and effective treatments against psoriasis.

## Figures and Tables

**Table 1:** Accepted topical treatments for psoriasis.

Active Ingredient	Effects	Drawbacks	References
Moisturizers	Reduces hyperproliferation, differentiation, and apoptosis. Moreover, anti-inflammatory effects and improving barrier function.	Irritant dermatitis, allergic contact dermatitis, fragrance allergy, stinging, and acne.	[67,68]
Coal Tar	Suppresses DNA synthesis, reducing the hyperproliferation of keratinocytes.	Odour, staining, irritant contact dermatitis, erythema, stinging, folliculitis, and formation of keratoacanthomas.	[69]
Salicylic acid	Reduces intercellular cohesiveness of the horny cells by dissolving the intercellular cement material. Furthermore, it reduces the pH of the stratum corneum, increasing hydration and softening.	Potential chronic or acute systemic intoxication, oral mucosa burning, frontal headache, central nervous system symptoms, metabolic acidosis, tinnitus, nausea, and vomiting.	[70]
Topical calcineurin inhibitors (TCIs)	It inhibits the action of calcineurin phosphatase and block the production of inflammatory substances that are thought to be important in causing skin lesions.	Stinging sensation and skin irritation.	[71]
Tazarotene	It binds to β and γ retinoic acid on the cell membrane of keratinocytes and is then transported to the nucleus, altering transcription of genes in keratinocytes.	The most common side effect of tazarotene is localized irritation.	[72,73]
Anthralin (Dithranol)	It reduces keratinocyte proliferation, prevents T-cell activation, and restores cell differentiation, probably through mitochondrial dysfunction.	Skin irritation, stains lesioned, and adjoining skin, hair, nails, clothing, and other objects, with which the patients come into contact.	[74]
Topical corticosteroids	Corticosteroids are vasoconstrictive, antiproliferative, anti-inflammatory, and immunosuppressive. They bind to the intracellular corticosteroid receptor and regulate gene transcription of numerous genes, particularly those that code for proinflammatory cytokines.	Skin atrophy striae, telangiectasia, or secondary infection. Therefore, potent TCS should not be used on the face or intertriginous sites. Systemic adverse events occur when TCS is used for prolonged periods of time or at doses higher than commonly prescribed. Prolonged use of potent TCS may result in its significant systemic absorption, which can lead to HPA axis suppression, Cushing’s syndrome, and hyperglycaemia.	[36,75]
Vitamin D analogues	Vitamin D analogues bind to the intracellular Vitamin D receptor, which then binds to and regulates the genes involved in epidermal proliferation, inflammation, and keratinization.	Skin irritation, hypercalcemia, hypercalciuria, and parathyroid hormone suppression, but these are very rare.	[76]

**Table 2:** Accepted systemic treatments.

Type of Treatment	Drug	Effects	Drawbacks	References
Conventional treatments	Acitretin	It binds to nuclear receptors on genes controlling cellular differentiation, anti-proliferation, anti-inflammation, anti-keratinization, and inhibition of neutrophil chemotaxis. It is the only systemic treatment that is not immunosuppressive.	Depression, hypertriglyceridemia and hypercholesterolemia, Myalgias, cheilitis, skin peeling, alopecia, xerosis, rhinitis, nail dystrophy, epistaxis, sticky skin, retinoid dermatitis, and xerophthalmia.	[77]
	Fumaric Acid Esthers (FAEs)	It has immunomodulatory, anti-inflammatory, and antiproliferative properties and apoptotic actions on activated T cells.	Warmth, reddening of the face, and headaches, proteinuria, reversible renal insufficiency, microscopic haematuria, and proximal tubular damage.	[78]
	Cyclosporine	It inhibits Interleukin synthesis, such as IL-2 and T cell differentiation.	Hypertension, arrythmia, hypertension, anxiety, headaches, fever, hypomagnesemia, hyperkalaemia, dyslipidaemia, and encephalopathy.	[79]
	Methotrexate	It reduces interleukin (IL)-17 mRNA and IL-17 protein expression in CD3- and CD28-stimulated peripheral blood mononuclear cells. It modulates pro-inflammatory mediators and its effects on atherogenic gene expression in psoriatic lesion skin.	Nausea, stomach pain, and diarrhoea.	[80]
Small molecules	Apremilast	It inhibits the expression and/or production of TNF-α, IFN- c, IL-12, and IL-23 and the chemokines CXCL9, CXCL10, CCL2, and CCL3, IL-2, IL-5, IL-13, IL-17, TNF-α, and IFN-c by stimulated T cells and IFN-a by dendritic cells.	Nausea, diarrhoea, and headaches.	[81]
Small molecules	Tofacitinib	It is a potent inhibitor of JAK1 and JAK3 and has some activity against JAK2 and Tyk2.	Nasopharyngitis, upper respiratory tract infection, headache, urinary tract infection, and diarrhoea.	[82]
Anti-TNF alpha	Infliximab	It interferes with the actions of TNF-α by directly binding to soluble and transmembrane TNF-α molecules in the plasma and the diseased tissue.	Dyspnoea, urticaria, hypotension, flushing, and headache.	[83]
	Etanercept	It is a recombinant human TNF-recipient by subcutaneous injection. Tor fusion protein that antagonizes the effects of endogenous TNF by competitively inhibiting its in- study disintegration with cell-surface receptors.	Respiratory infections, flu-like symptoms, and gastrointestinal symptoms.	[84,85]
	Adalimumab	It blocks its interaction with the p55 and p75 cell surface TNF receptors.	Headache, nausea, elevated triglycerides, cough, sinus congestion, and fatigue most common.	[86]
	Certolizumab	It inhibits lipopolysaccharide-induced IL-1-β release from monocytes and provokes nonapoptotic cell death in tmTNF- α-expressing cells.	Headache, nasopharyngitis, upper respiratory tract infections, diarrhoea, and sinusitis.	[87,88]
Anti-IL12/23	Ustekinumab	It is a human monoclonal antibody that binds to the shared p40 protein subunit of human interleukins 12 and 23 with high affinity and specificity (unpublished data), thereby preventing interaction with their cell surface IL12Rβ1 receptor.	Headache, nasopharyngitis, arthralgia, and upper respiratory tract system.	[89]
Anti-IL17	Secukinumab	It is a recombinant, high-affinity, fully human immunoglobulin G1κ monoclonal antibody that selectively binds and neutralizes interleukin-17A.	Nasopharyngitis, headache, and upper respiratory tract infection.	[90]
	Bimekizumab	It is a monoclonal antibody of the immunoglobulin G1 isotype, rationally designed to be able to bind at a similar site on both IL-17A and IL-17F, conveying dual inhibition of both isoforms.	Nasopharyngitis, oropharyngeal pain, and headache.	[91]
	Ixekizumab	It is recombinant, high-affinity, humanized IgG4-κ monoclonal antibody, which selectively binds and neutralizes interleukin 17A (IL-17A), the proinflammatory and primary effector cytokine of type 17 helper T (Th17) cells.	Nasopharyngitis, upper respiratory infection, injection-site reaction, and headache.	[92,93]
IL17 R antagonist	Brodalumab	It binds with high affinity to human interleukin-17RA and blocks the biologic activity of interleukins 17A, 17F, 17A/F heterodimer, and 17E (interleukin-25).	Nasopharyngitis, upper respiratory tract infection, arthralgia, and erythema at the injection site.	[94]
Anti-IL23	Tildrakizumab	It is a novel, high-affinity humanized IgG1/j monoclonal antibody that specifically binds to the p19 subunit of human IL-23 without binding IL-12.	Nasopharyngitis and headache.	[95]
	Guselkumab	It is a fully human IgG1 lambda monoclonal antibody that binds to the p19 subunit of IL-23.	Nasopharyngitis and upper respiratory tract infection.	[96,97]
	Risankizumab	It is a humanised IgG1 monoclonal anti- body that binds the p19 subunit of IL-23, thus inhibiting this key cytokine and its role in psoriatic inflammation.	Upper respiratory tract infection, urinary tract infection, influenza, and headache.	[98]
	Mirikizumab	It is a humanized IgG4-variant monoclonal antibody that binds to the p19 subunit of IL-23 and does not bind IL-12.	Viral upper and other respiratory tract infections, injection-site pain, hypertension, and diarrhoea.	[99]

**Table 3:** Cutting edge treatments for psoriasis under clinical trials.

Clinical Trial Phase	Drug Name	Administration Via	Targeting	References
Phase 1	BOS-475	Topical	Bromodomain and extraterminal domain protein inhibitors	[101]
	ABBV-157	Oral	RORγt inhibitor	[102]
	CC-92252	Oral	Interleukin-2 receptor agonists; Regulatory T-lymphocyte stimulants	[103]
	EDP 1066	Oral	Immunomodulators	[104]
	EDP 1815	Oral	Immunomodulator	[105]
Phase II	ABY-035	Parenteral	IL-17A inhibitor	[106]
	ARQ-151	Topical	PDE4 Enzyme inhibitor	[107]
	BI 730357	Oral	Nuclear receptor antagonist	[108]
	EISO (SAN021)	Topical	PDE4 blocker	[109]
	JTE-451	Oral	ROR inhibitor	[110]
	M1095	Parenteral	Trivalent monomeric nanobody that neutralizes interleukins IL-17A, IL-17F, and IL-17A/F	[111]
	PF-06700841	Topical	JAK1 and TYK2 inhibitor	[112]
Phase II	PF-06826647	Oral	TYK2 inhibitor	[113]
	SHR-1314	Parenteral	IL-17A Antagonist	[114]
Phase III	BMS- 986165	Oral	Tyk2 inhibitor	[115]
	BCD-085	Parenteral	IL-17 inhibitor	[116]
	BI695502	Parenteral	TNF-α inhibitor	[117]
	CF101	Oral	Adosine A3 receptor inhibitor	[118]
	CHS-1420	Parenteral	TNF-α inhibitor	[119]
	Filgotinib	Oral	JAK 1 inhibitor	[120]
	Mirikizumab	Parenteral	IL-23 inhibitor	[121]
	Serlopitant	Oral	Neurokinin-1 receptor antagonist	[122]
	Tapinarof	Topical	AHR agonist	[123]
	Tikdrakizumab	Parenteral	IL-23 inhibitor	[124]
	Upadacitinib	Oral	JAK inhibitor	[125]

**Table 4:** Biodegradable nanotechnology-based treatments for psoriasis.

Nanocarrier	Advantages	Limitations	Drug Released	Administration Via	References
Nanospheres	Enhanced solubility, extended release of drug, and improve absorption.	Poor drug loading, agglomeration, storage issues, problems in large scale production.	Vitamin D3	Topical	[128]
			Bethamethasone bisodium 21-phosphate	Intravenous	[157]
Nanocapsules	Improved skin permeation, sustained and controlled release, improved selectivity, and biocompatibility.	Poor drug loading, agglomeration, storage issues, problems in large scale production.	Tretinoin	Topical	[158]
			Dexamethasone	Topical	[131]
Dendrimers	Ease of preparation and modification.	Polymer dependent biocompatibility.	TNF-α Si RNA	Topical	[159]
			Dithranol	Topical	[134]
Micelles	Self-assembling, thermodynamic, stability, and targeting potential.	Not good for hydrophilic drugs.	Tacrolimus	Topical	[136]
			Cyclosporine A	Topical	[160]
Liposomes	Biocompatible, ease of surface modification, and amphiphilic nature.	Weak loading capacity, rapid drug leakage, limited physical and chemical stability during storage.	Fusidic acid	Topical	[140]
			Methotrexate	Topical	[161]
			Cyclosporine	Topical	[162]
			Calcipotriol	Topical	[163]
Lipospheres	Biocompatible, amphiphilic nature, and surface modification is easy.	Weak loading capacity, rapid drug leakage, limited physical and chemical stability during storage.	Tacrolimus and curcumin	Topical	[142]
Ethosomes	Very good permeation power, high patient compliance. Composition is safe for dermal and pharmaceutical use.	Poor yield	Cyclosporine	Topical	[164]
			Psoralen	Topical	[144]
			Tacrolimus	Topical	[165]
Solid lipid nanoparticles	Biocompatible, biodegradable, higher efficacy, flexibility of size, and surface manipulation	Poor stability, poor batch to batch reproducibility, sterilization difficulties, and low drug loading	Fluocinolone acetonide	Topical	[149]
			Capsaicin	Topical	[166]
			Betamethasone dipropionate and Calcipotriol	Topical	[167]
Nanostructured lipid carriers	Biodegradable, biocompatible, reduces expulsion of drug during storage, good drug load	Sterilization difficulties	Methotrexate	Topical	[153]
			Fluticasone propionate	Topical	[168]
			Calcipotriol and methotrexate	Topical	[169]

## Data Availability

Not applicable.
